# Dramatic enhancement of supercontinuum generation in elliptically-polarized laser filaments

**DOI:** 10.1038/srep20363

**Published:** 2016-02-05

**Authors:** Shermineh Rostami, Michael Chini, Khan Lim, John P. Palastro, Magali Durand, Jean-Claude Diels, Ladan Arissian, Matthieu Baudelet, Martin Richardson

**Affiliations:** 1 Center for High Tech Materials, University of New Mexico, Albuquerque NM 87106, USA.; 2 Townes Laser Institute, CREOL – The College of Optics and Photonics, University of Central Florida, Orlando FL 32816, USA; 3 Plasma Physics Division, Naval Research Laboratory, Washington, DC 20375-5346, USA; 4 Department of Physics, University of Central Florida, Orlando FL 32816, USA; 5 National Center for Forensic Science, University of Central Florida, Orlando FL 32816, USA.; 6 Department of Chemistry, University of Central Florida,Orlando FL 32816, USA

## Abstract

Broadband laser sources based on supercontinuum generation in femtosecond laser filamentation have enabled applications from stand-off sensing and spectroscopy to the generation and self-compression of high-energy few-cycle pulses. Filamentation relies on the dynamic balance between self-focusing and plasma defocusing – mediated by the Kerr nonlinearity and multiphoton or tunnel ionization, respectively. The filament properties, including the supercontinuum generation, are therefore highly sensitive to the properties of both the laser source and the propagation medium. Here, we report the anomalous spectral broadening of the supercontinuum for filamentation in molecular gases, which is observed for specific elliptical polarization states of the input laser pulse. The resulting spectrum is accompanied by a modification of the supercontinuum polarization state and a lengthening of the filament plasma column. Our experimental results and accompanying simulations suggest that rotational dynamics of diatomic molecules play an essential role in filamentation-induced supercontinuum generation, which can be controlled with polarization ellipticity.

Supercontinuum generation (SCG) resulting from laser filamentation in gases, was first observed 20 years ago^1^,^2^, and has since attracted the interest of scientists in diverse fields of research. It has proven critical to the understanding of laser-matter interactions underlying filamentation, the generation and compression of few-cycle pulses^3^,^4^,^5^, and the generation of secondary sources for sensing (LIDAR)^6^ and countermeasure applications, for instance. Despite this long history, there remains a need for more complete understanding of the mechanisms underlying filament-induced SCG. Numerous factors appear to play a significant role in filament SCG, including the chirp^7^,^8^,^9^ and polarization^10^,^11^ of the input laser pulse, as well as control of the focusing process before filamentation by preparation in the medium^12^,^13^ or the focusing conditions^13^.

In this article, the effect of the laser polarization on filamentation and the consequent SCG is studied. Despite clear understanding of SCG generated by linear polarization, there remains controversy when it comes to circular polarization. Different studies (under various experimental or simulated conditions) have reported circularly polarized filaments to be more or less efficient for SCG^11^,^15^,^16^,^17^, as well as plasma formation^10^,^17^. Varela *et al*.^18^ showed that independent of the nature of the gas, increasing the ellipticity from linear to circular induces a decrease in spectral bandwidth and suppression in the appearance of multiple filaments, therefore allowing more energy to be channeled into a single filament under the right conditions. Sandhu *et al*.^11^ explored the ellipticity dependence of SCG in molecules with longer pulses and observe a dependence on the molecular structure with the same trend of enhanced broadening for linearly polarized light.

The polarization state of the light itself has been reported to be more stable^19^ or less stable^20^ after filamentation of circularly polarized input pulses. Recently it was measured that the light’s polarization state changes during the propagation of an intense elliptically polarized beam in air. This change of polarization was found to vary across the beam profile and was dependent on the medium^21^. Simulations have since shown that the delayed Kerr response due to molecular alignment is responsible for polarization modification in filament propagation^22^.

Here, we present a combined experimental and theoretical study of the supercontinuum spectrum created by filamentation of elliptically polarized laser pulses along with polarization measurement of the laser beam after filamentation. Our measurements show an enhancement of the supercontinuum bandwidth generated by the filamentation of pulses with specific polarization ellipticities in molecular gases. The enhancement of the supercontinuum generation coincides with input light ellipticities that experience the largest polarization changes. Our experimental results and accompanying simulations suggest that rotational dynamics of the diatomic molecules play an essential role in the filamentation-induced SCG, which can be controlled with light ellipticity.

We measure the spectrum and polarization ellipticity of the supercontinuum generated through filamentation of a femtosecond laser pulse (50 fs, 2.8 mJ)^23^ in different gases and for different prepared input polarization ellipticities. The input ellipticity is controlled by rotating a broadband quarter wave plate (QWP) before filamentation is aided by low numerical aperture (NA ≈ 10^−3^) focusing^14^ (see Fig. 1a). In nitrogen, we observe a dramatic anomalous broadening of the supercontinuum spectrum (Fig. 1b) for quarter wave plate angles of 39 and 51 degrees (input ellipticity *ε*_*in*_ ≈ 0.7). In oxygen (see Supplementary Fig. 1) the broadest spectra appear for QWP angles of 43 and 47 degrees (*ε*_*in*_ ≈ 0.8). The ellipticity of the beam (ratio of the minor to major axis of the polarization ellipse) after filamentation in nitrogen is plotted in Fig. 1c as a function of the QWP angle. It is clear that propagation of the filament significantly modifies the polarization at the same angles that produce enhanced spectral broadening. Figure 1d,e show no anomalous polarization dependent features for the same measurements performed in argon, which has a comparable ionization potential to nitrogen (15.76 eV and 15.58 eV, respectively).

Comparing the supercontinuum spectrum generated by a single filament in atomic (argon and krypton) and molecular gases (nitrogen, oxygen and air) (see Fig. 1 and Supplementary Fig. 1), the observed anomalous behavior is evidently unique to filament-induced SCG in molecular gases. In atomic gases we observe broader white light spectrum for linear polarization than for circular polarization, with the bandwidth decreasing monotonically with increasing ellipticity^24^. In molecular gases however, for a specific input ellipticity, spectrum extends well beyond the shortest wavelengths observed with linear input polarization, while the output ellipticity exhibits a remarkable modification. In every case, the behavior is symmetric about circular polarization, indicating that the broadening is identical for left- and right-handed elliptical polarizations.

Previous experiments with elliptically polarized pulses have also shown anomalous behavior in the polarization of the beam after filamentation in air^20^,^21^. Under comparable conditions (60 fs, 5 mJ pulses inducing single filamentation in air) to the experiments presented here, substantial modification of polarization was observed at similar input ellipticity close to circular polarization^21^. However no unusual behavior was observed for filaments prepared under vacuum focusing conditions^13^ (See Supplementary Fig. 2), which shows that the nonlinear focusing (involving the Kerr response of the molecular medium) is necessary for the observation of anomalous behavior in filaments induced by elliptically polarized pulses.

The peculiar behavior described above must be the result of polarization-induced modifications to the filament properties. Indeed, we observe an unexpected lengthening of the filament in molecular gases, which is correlated to the spectral broadening and polarization modification. To measure the filament length, transverse plasma emission from N_2_ is imaged with a camera as the angle of the quarter wave plate is changed. The results shown in Fig. 2 indicate that the collapse position gradually moves away from the lens as the polarization changes from linear to circular. This is consistent with the reduced strength of χ^(3)^ effects for circular as compared to linear polarization, resulting in a weaker self-focusing and delaying the onset of filamentation^9^. However, for the QWP angle of 39 degrees, the filament plasma channel is observed to extend further from the focusing lens, indicating that the self-focusing and plasma defocusing are balanced over a longer distance.

It is well known that molecular rotation can play a significant role in filamentation, with alignment-induced birefringence^25^, broader supercontinuum spectra^26^ and longer plasma channels^27^ observed for filamentation in pre-aligned media. However, determining the role of the rotational response in a single pulse remains challenging for both experimentalists and theorists. Early modeling of nonlinear propagation in molecular media by Close^28^, indicated that for elliptical polarization, left- and right-handed circularly polarized components of the field experience different nonlinear indices of refraction. Later modeling by Kolesik *et al*.^17^, which included the vector nature of the Kerr nonlinearity (i.e. cross- as well as self-Kerr effects), losses due to ionization, and the delayed nonlinear susceptibility associated with molecular rotation, suggested a more complex behavior of the polarization after filamentation. For an elliptically polarized input pulse, the filamentation process resulted in a more circularly polarized output in the filament core, without indication of a sudden broadening of the supercontinuum or modification of the polarization. However, even this more realistic treatment may significantly underestimate the coupling between different polarization components due to simplified classical modeling of the delayed molecular rotational response.

In this study, theoretical modeling of filamentation in molecular gases was performed by including a self-consistent linear density matrix treatment of the rotational dielectric response of the molecules subjected to arbitrary light polarizations in a nonlinear propagation model (see Methods). Previous simulations carried out under the same model^22^, indicated that strong coupling of left- and right-handed circular polarization states arises from off-diagonal terms in the molecular susceptibility tensor, resulting in similar anomalous behavior to what we observe in the experiments. Simulated and experimentally measured supercontinuum spectra resulting from filamentation in nitrogen and oxygen gases are compared in Fig. 3. The qualitative agreement between experiments (Fig. 3a,c) and simulations (Fig. 3b,d) carried out under identical conditions, along with the fact that there is no anomalous behavior in atomic gases (Supplementary Fig. 3), indicate that the necessary physics for describing the anomalous behavior is included in the rotational contribution to the molecular susceptibility. The main physical mechanism implied by our simulation is a polarization dependent pulse shaping, resulting in a second self-focusing event and pulse self-steepening at particular elliptical polarizations. More theoretical work is required to match the range of ellipticities for which enhanced SCG is observed, and the particular value of ellipticity for which optimal enhancement is observed in each gas. Other mechanisms such as ellipticity dependencies in the ionization rates or population of excited states could also be involved in explanation of the ellipticity dependent filamentation process^29^, and are not accurately taken into account in the simulations.

The simulated results allow us to investigate more carefully the spatio-temporal evolution of the filamenting pulse for different input ellipticities. In Fig. 4, we show the on-axis peak intensity and electron density for filaments created in N_2_ from pulses with input ellipticities *ε*_*in*_ = 0.4, 0.7 and 1. As mentioned above, the reduced strength of both χ^(3)^ effects and multiphoton ionization for larger values of ellipticity results in a higher on-axis peak intensity at the filament collapse location (Fig. 4a, *z* ≈ 250 cm after the focusing lens), and in turn a higher electron density (Fig. 4b), for smaller values of ellipticity. Interestingly, however, for the input ellipticity *ε*_*in*_ = 0.7, a second self-focusing event occurs (*z* ≈ 350 cm), reaching higher values of both on-axis intensity and electron density than at the initial collapse location. Experimentally, this phenomenon was observed not as a secondary plasma channel, but instead as an elongation of the initial plasma channel. Only for the input ellipticity *ε*_*in*_ = 0.7, we observe a significant self-steepening of the pulse following the second nonlinear focus, resulting in the dramatic broadening of the supercontinuum spectrum (Fig. 4d) and the modification of the supercontinuum polarization state for a particular value of input ellipticity (see Supplementary Fig. 4).

The second self-focusing event can be explained by coupling between the left- and right-handed circular polarization states. Our simulations indicate that energy exchange between the two states tend towards an equal energy sharing between left- and right-handed circular polarization, with the dominant polarization state transferring energy into the weaker polarization state during propagation (see Supplementary Fig. 5). The different self-focusing and ionization for different initial ellipticities result in conditions which are more or less favorable for a second self-focusing event: *(1)* For small input ellipticities, the initial nonlinear focus is strong, creating a large amount of plasma which in turn strongly refracts the pulse. The high intensity allows rapid energy equilibration between the two polarization states; however any further refocusing is diminished because of the large refraction. *(2)* For intermediate ellipticities, the initial nonlinear focus is more mild, allowing the dominant polarization state to refocus and therefore to strengthen the coupling between the two polarization states. For *ε*_*in*_ = 0.7, we find that the energy of left- and right-handed circularly polarized states is nearly equal following this second self-focusing event, resulting in a reduction of the measured output ellipticity. *(3)* For large ellipticities, the initial nonlinear focus is significantly weaker, and the refocusing is prevented by the small nonlinear index for near-circularly polarized pulses.

The difference in the optimal input ellipticity for SCG in N_2_ and O_2_ gases can be explained by the coupling strength between left- and right-handed circular polarizations. This coupling is determined (see Methods) by the off-diagonal components of the susceptibility matrix 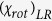, which are proportional to 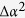, where 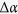 is the difference in the molecular polarizability parallel and perpendicular to the molecular axis. Therefore, larger 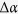 results in more coupling between polarization states, and a larger disparity between left- and right-handed circular polarization states can be “corrected” during propagation. Thus, for larger 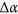 we expect that the optimal input ellipticity for SCG will be closer to circular polarization, where there is a larger imbalance between the left- and right-handed components. In the cases studied here, O_2_ has a larger value of 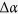 than N_2_, and we observe enhanced SCG in O_2_ for a larger value of input ellipticity.

In summary, we have demonstrated a dramatic enhancement in the spectral broadening of filament-induced SCG, which can be controlled by tuning the initial ellipticity of the laser pulse undergoing filamentation. This effect, which is observed only for molecular gases, is correlated to a decrease in the ellipticity of the beam after filamentation and an increase in the length of the filament plasma channel. Both experimental and simulated results clearly demonstrate the strong dependence of filamentation on the input laser ellipticity via the delayed rotational response of the molecular medium. This study introduces an additional tool – polarization ellipticity – to control SCG and enhance the white light spectrum and suggests that further refinements of filamentation models may provide a route to better understanding the dynamic of laser-matter interaction underlying filament propagation.

## Methods

### Supercontinuum generation and diagnostic

**Figure 1:**
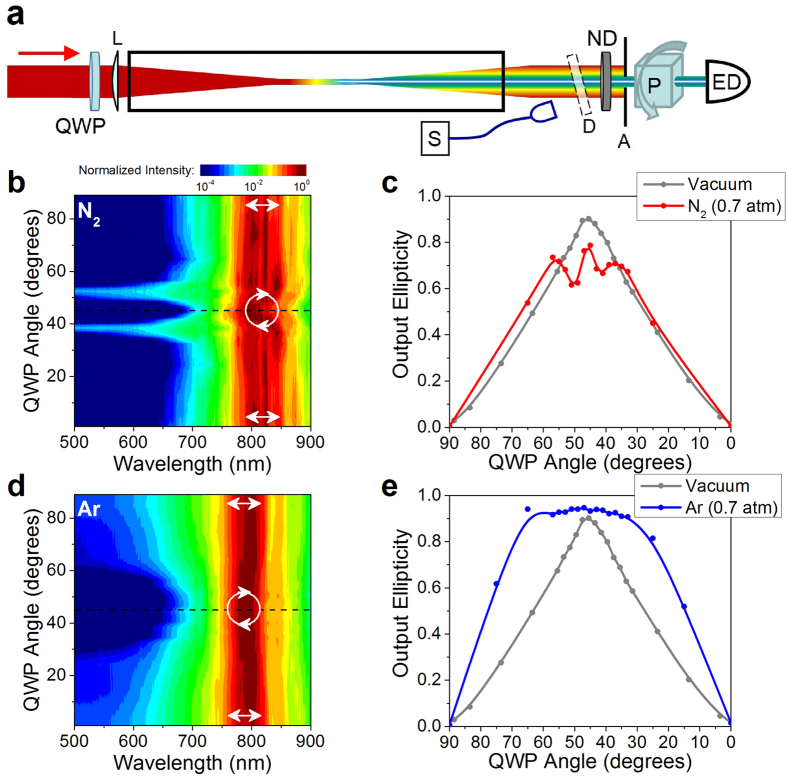
Anomalous behavior in supercontinuum and polarization. **(a)** Experimental setup to measure the supercontinuum spectrum and polarization ellipticity. The 50 fs, 2.8 mJ pulses at 800 nm are focused by a lens (f = 3 m) into a 4.5 m gas chamber. A quarter wave plate (QWP) is rotated to prepare the beam with different initial elliptical polarization. After attenuation by a neutral density filter (ND), the central part of the beam is selected by an aperture (A). A rotating polarizer cube and an energy detector (ED) are then used to record the polarization ellipse. A fiber-coupled spectrometer (S) records the scattering from a diffuser (D). (**b)** Spectral intensity of the supercontinuum spectrum as a function of wavelength and QWP angle measured in nitrogen (normalized to highest value in 2D map). White arrows indicate the angles for linear and near-circular polarization. The wavelength for which the power spectral density drops to 1% of its maximal value is found to be 725 nm for QWP angles of 0 and 90 degrees, and 685 nm for 39 degrees. (**c)** Ellipticity measured after the filament (red) created in nitrogen and with the chamber evacuated (gray) as a function of the quarter wave plate angle. **(d,e)** Same measurements as in **(b,c)** for filaments in argon gas.

In the experiments, femtosecond laser pulses (pulse duration *τ*_*p*_ = 50 fs, energy *E* = 2.8 mJ, wavelength *λ* = 800 nm) from the Multi-Terawatt Femtosecond Laser (MTFL) facility^23^ at the University of Central Florida were focused by a lens (focal length *f* = 3 m), into a transparent gas-filled chamber (length *L* = 4.5 m). The length of the chamber, and the thickness of the SiO_2_ windows (2.0 mm at input and output) were chosen to prevent the effects of nonlinearity in the window. This was checked by comparing measurements of filamentation in air with and without the windows on the chamber, for which no difference was observed. The experimental set-up is shown in Fig. 1a. Elliptically polarized laser pulses, created using a zero order QWP placed just before the focusing lens, were used to produce single (through pressure control) filaments individually in air, nitrogen (N_2_), oxygen (O_2_), argon, and krypton. The spectrum of the white light supercontinuum after filamentation was measured after scattering by a diffuser, using spectrometers covering visible (λ = 300–740 nm, Ocean Optics HR2000) and near-infrared (λ = 660–930 nm, Ocean Optics USB2000) regions of the spectrum. Polarization of the beam after filamentation was determined by fitting the polarization ellipse obtained by measuring the transmitted energy (attenuated using a neutral density filter) through a rotating polarizing cube, for each QWP angle. For both the spectral and polarization measurements, the detectors were placed approximately 2.2 meters after the geometric focus of the lens.

### Simulations

The laser pulse evolution and supercontinuum generation are simulated using the modified paraxial wave equation in azimuthally symmetric, cylindrical coordinates. The transverse vector electric field, 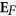, is expressed as an envelope 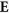, modulated by phase: 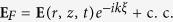, where the pulse envelope evolves according to:


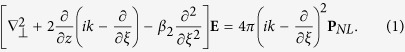


In Eq. (1) 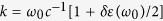, 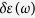 is the shift in dielectric constant due to linear dispersion, 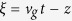 is the group velocity frame coordinate, 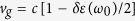, and 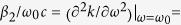 20 fs^2^/m accounts for group velocity dispersion. The nonlinear polarization density, 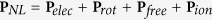, includes the instantaneous (Kerr) response, the delayed molecular rotational response, the free electron response, and ionization energy losses respectively^22^,^30^.

The electronic and rotational polarization densities can be expressed as the product of an effective susceptibility matrix and the electric field envelope: 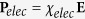 and 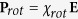. Using the circularly polarized basis (with subscripts L: left-handed and R: right-handed circular polarization), the effective susceptibility matrix elements are given by


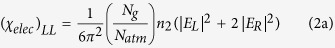



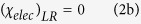



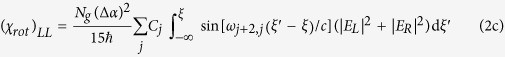



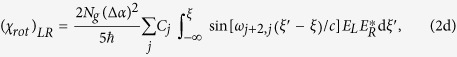


where


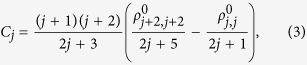



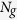
 is the gas density, 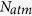 is the gas density at 1 atm, 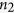 is the instantaneous Kerr coefficient at 1 atm^31^, 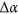 is the molecular polarizability anisotropy^32^, 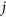 is the total angular momentum quantum number, 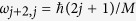, 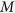 is the molecular moment of inertia, 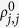 are the thermal equilibrium density matrix elements, and the remaining matrix elements can be obtained via L-R suffix interchange^21^. The values of 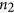 and 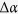 are taken from refs. 31 and 32, respectively, and are given in Table 1. Rotational states up to 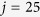 were included in the sums. The free electron and ionization damping polarization densities are determined by 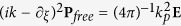 and 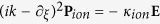, where:


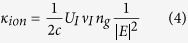


is the damping rate, 
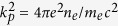
 is the plasma wavenumber, 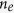 is the free electron density, 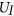 is the ionization potential, 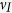 the cycle averaged ionization rate^22^,^33^, and 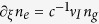. Additional details about the response model can be found in ref. 22.

## Additional Information

**How to cite this article**: Rostami, S. *et al*. Dramatic enhancement of supercontinuum generation in elliptically-polarized laser filaments. *Sci. Rep*. **6**, 20363; doi: 10.1038/srep20363 (2016).

## Supplementary Material

Supplementary Information

## Figures and Tables

**Figure 2:**
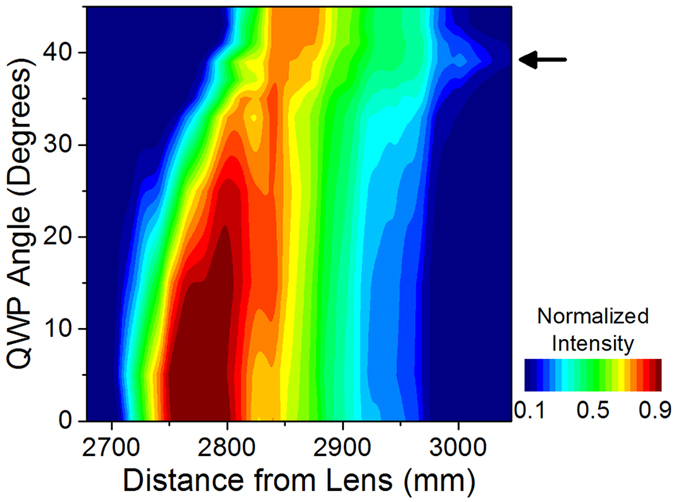
Ellipticity dependence of filament plasma channel. The experimentally measured intensity of the transverse plasma emission in N_2_ is plotted for various QWP angles, exhibiting an anomalous extension away from the focal lens when the QWP angle is set to 39 degrees, as indicated by the arrow. The length of the filament plasma channel (characterized by the distances at which the plasma emission intensity drops to 1/*e*^2^ of its maximal value) with the QWP angle set to 39 degrees (290 mm) is found to be more than 30% longer than for 45 degrees (220 mm) and more than 5% longer than for 0 degrees (270 mm).

**Figure 3:**
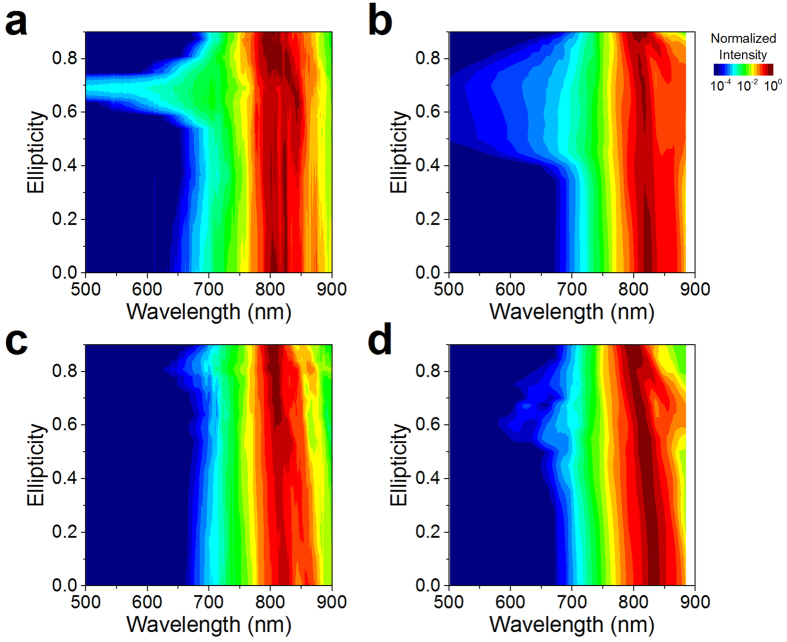
Dependence of supercontinuum spectra on the input ellipticity. Experimental (**a**,**c**) and simulated (**b**,**d**) results are compared for single filaments in nitrogen (**a**,**b**) and oxygen (**c**,**d**) gases. Experiments and simulations (See Supplementary Fig. 3) in atomic gases (Ar and Kr), showed no anomalous behavior.

**Figure 4:**
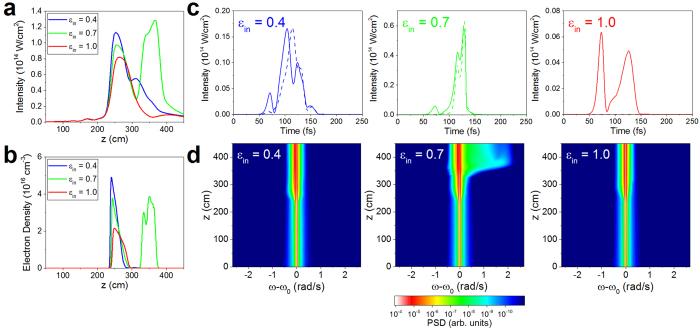
Dependence of the on axis intensity, electron density, and supercontinuum generation on propagation and initial ellipticity. (**a**) Simulated peak intensity and (**b**) on axis electron density for initial polarizations of *ε*_*in*_ ≈ 0.4 (blue) *ε*_*in *_≈ 0.7 (green) and *ε*_*in*_ ≈ 1 (red), in Nitrogen. The distance 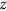 is measured from the focusing lens, with the geometrical focus located at 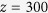 cm. (**c**) Time-dependent intensities of right- (solid lines) and left-handed (dashed lines) circular polarization components for input ellipticities of 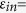 0.4, 0.7, and 1. 0 at 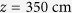 Strong self-steepening is observed for 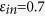. (**d**) Evolution of the supercontinuum power spectral density (PSD) with propagation distance. At the location of the second nonlinear focus (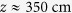), a large shift of the spectrum to higher frequencies is observed.

**Table 1:** Nonlinear coefficients used in the simulations.

Gas species	*n*_*2*_ (10^−20^ cm^2^/W)	*Δα* (10^−25^ cm^3^)
N_2_	7.4	9.3
O_2_	9.5	11.4
