# Supplementary material for: Dramatic enhancement of supercontinuum generation in elliptically-polarized laser filaments
Source: Sci Rep. 2016 Feb 5;6:20363. doi: 10.1038/srep20363 (PMC4742882; doi:10.1038/srep20363)
Supplement: Supplementary Information [file srep20363-s1.pdf]

# Dramatic enhancement of supercontinuum generation in elliptically-polarized laser filaments

## (Supplementary Information)

Shermineh Rostami[1], Michael Chini[2], Khan Lim[2], John P. Palastro[3], Magali Durand[2], Jean-Claude Diels[1], Ladan Arissian[1], Matthieu Baudelet[2], and Martin Richardson[2]

[1]*Center for High Tech Materials, University of New Mexico, Albuquerque NM 87106*

[2]*Townes Laser Institute, CREOL – The College of Optics and Photonics, University of Central Florida, Orlando FL 32816, USA*

[3]*Plasma Physics Division, Naval Research Laboratory, Washington, DC 20375-5346, USA*

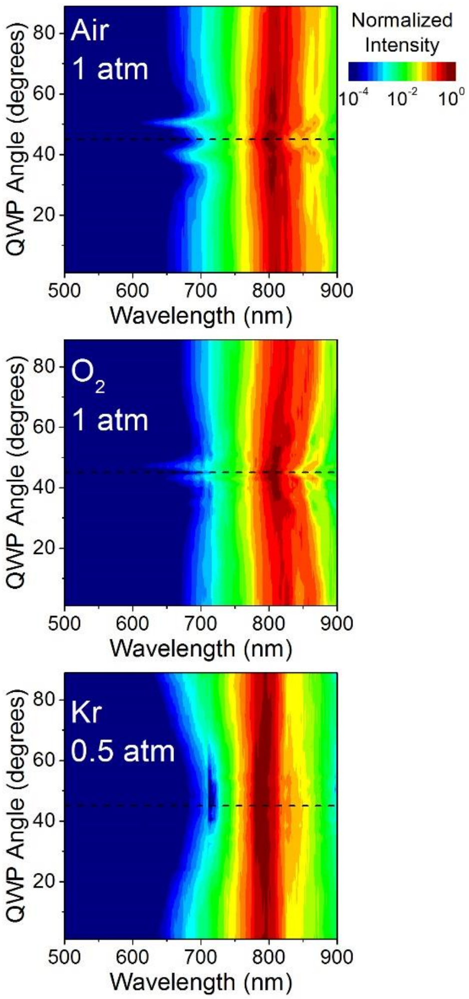


**Supplementary Fig. 1 |** Experimental results of ellipticity-dependent supercontinuum spectrum produced from single filaments in air, oxygen, and krypton gases.

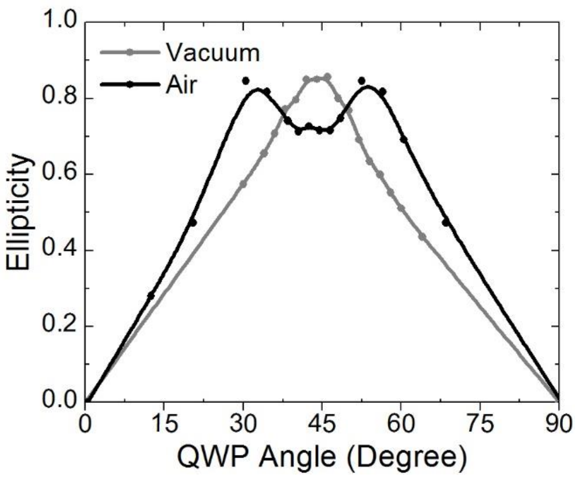


**Supplementary Fig. 2 |** Output ellipticity for air filaments prepared by focusing in vacuum (gray circles) and in air (black squares)

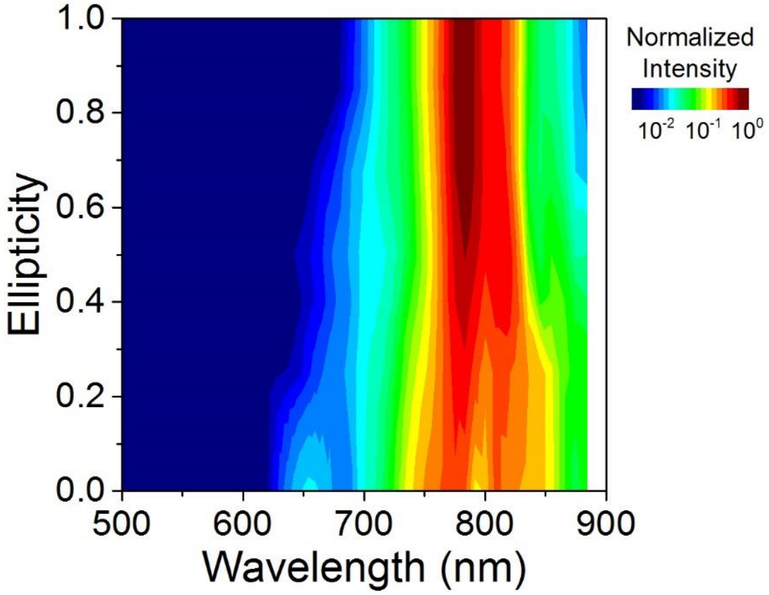


**Supplementary Fig. 3 |** Simulated ellipticity-dependent supercontinuum spectrum produced from single filaments in argon.

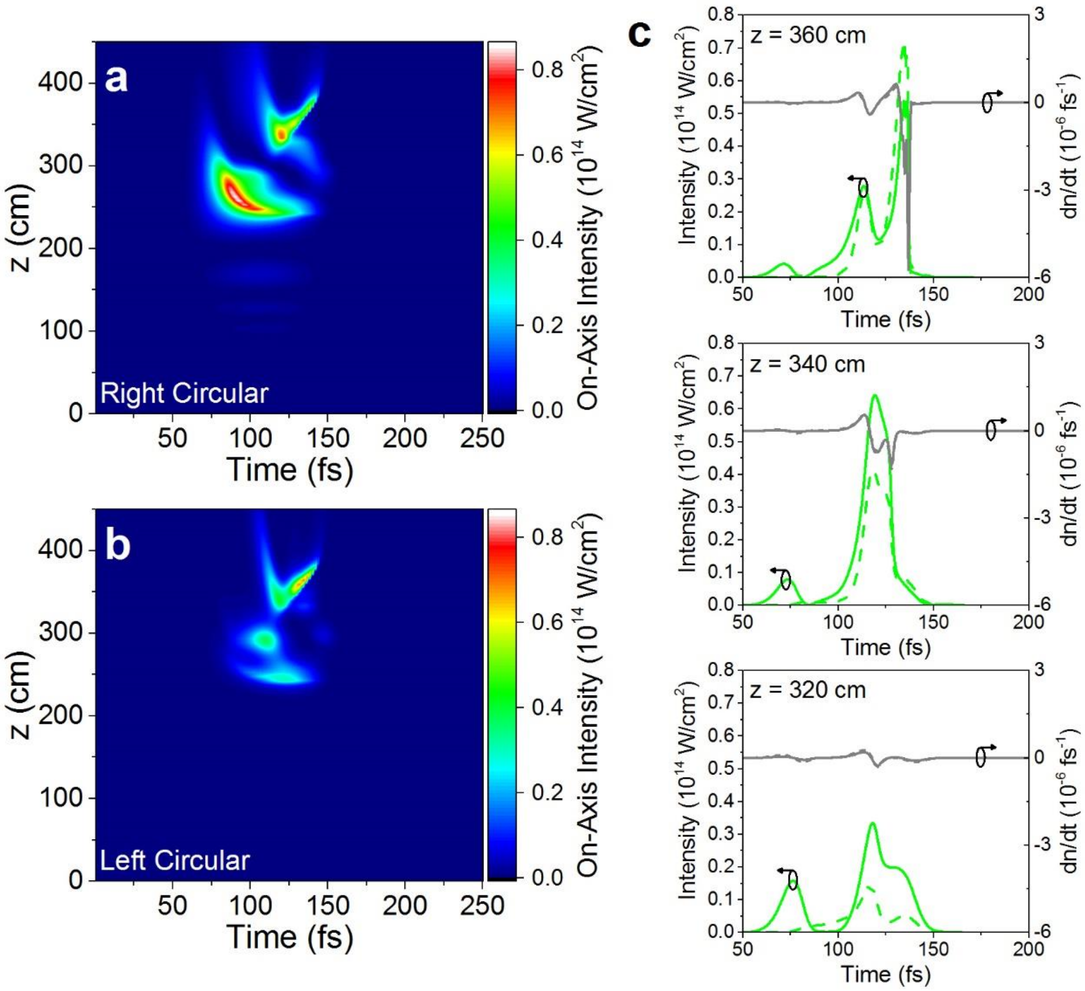


**Supplementary Fig. 4 |** On-axis intensity profiles during propagation for (**a**) the right- and (**b**) left-handed circularly polarized components for single filaments produced in nitrogen gas (0.7 atm), with $\varepsilon_{in} = 0.7$. Self-steepening of the pulse is apparent for $z > 300$ cm. **c,** Variation of the intensity (green) and refractive index with time ($\mathrm{d}n/\mathrm{d}t$, gray) for $z =$320, 340, and 360 cm. Right- and left-handed circular polarization components are indicated by solid and dashed lines, respectively. The negative values of $\mathrm{d}n/\mathrm{d}t$ at the trailing edge of the pulse at $z = 360$ cm are responsible for the blueshift of the spectrum.

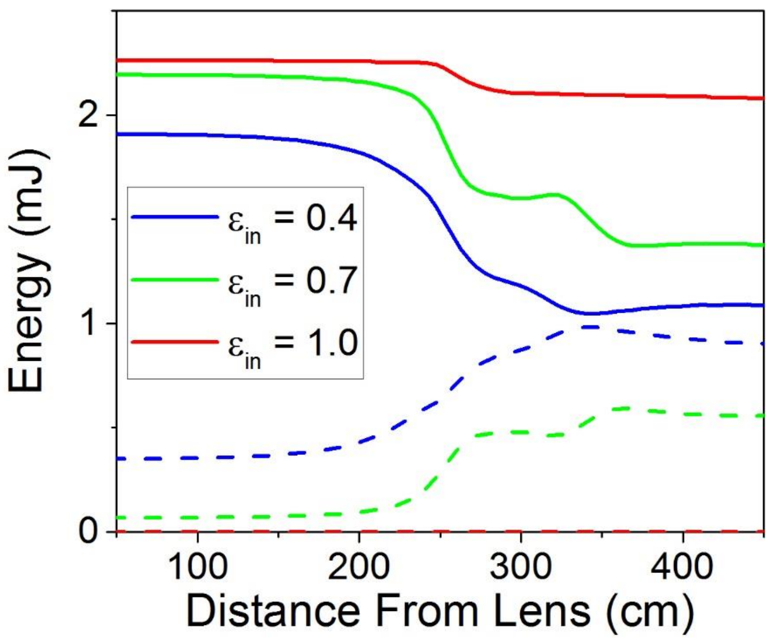


**Supplementary Fig. 5 |** Evolution of the right- (solid lines) and left-handed (dashed lines) circular polarization states with propagation for single filaments produced in nitrogen gas (0.7 atm). Note that for $\varepsilon_{in} = 0.4$ and $\varepsilon_{in} = 1.0$, energy equilibration occurs at the location of the initial nonlinear focus ($z \approx 250$ cm), while for $\varepsilon_{in} = 0.7$ a second equilibration step occurs at the second focus ($z \approx 350$ cm).
